# The effect of perceived organizational support on innovative behavior among nurses: a parallel mediation model of digital competence and work thriving

**DOI:** 10.3389/fpsyg.2026.1849118

**Published:** 2026-05-29

**Authors:** Qian Wu, Jie Liu

**Affiliations:** Department of Nursing, The First Hospital of Chongqing Medical University, Chongqing, China

**Keywords:** digital competence, innovative behavior, nurse, perceived organizational support, work thriving

## Abstract

**Background:**

With the deepening of digital transformation in healthcare, nurses’ innovative behavior has become a core driver for improving the quality of nursing services. While perceived organizational support is recognized as a key antecedent of innovative behavior, the mediating mechanisms of digital competence and work thriving in this relationship remain to be clarified.

**Methods:**

A cross-sectional survey was conducted among 414 clinical nurses. Measures included the Perceived Organizational Support Scale, Digital Competence Scale, Work Thriving Scale, and Innovative Behavior Scale. Based on organizational support theory, social cognitive theory, and social embeddedness theory, a parallel mediation model was constructed. Data were analyzed using SPSS with Hayes’ PROCESS macro (Model 4), and the mediating effects were tested via the bootstrap method.

**Results:**

The average innovative behavior score was 83.47 ± 12.44. Pearson correlation analysis showed that all four variables were significantly positively correlated with each other. Both digital competence and work thriving played significant parallel mediating roles. The indirect effect of digital competence was 0.549 (95% CI [0.446, 0.665]), accounting for 72.8% of the total effect, while the indirect effect of work thriving was 0.113 (95% CI [0.062, 0.173]), accounting for 15.0% of the total effect.

**Conclusion:**

Digital competence and work thriving serve as parallel mediators in the relationship between perceived organizational support and innovative behavior among clinical nurses. Cognitive and technical capacity may serve as a more direct and powerful factor of innovative behavior among clinical nurses, while positive psychological states provide important supplementary motivation. Targeted interventions to enhance perceived organizational support, improve digital competence, and promote work thriving are critical to fostering nurses’ innovative behavior and advancing high-quality nursing development.

## 1 Introduction

Over the past few years, rapid advances in digital healthcare and intelligent nursing development have placed increasingly higher demands on the professional ability of frontline nurses throughout the world (Phanphairoj et al., 2026; Lee et al., 2025; Schlicht et al., 2025). Among various core competencies, innovative behavior is regarded as a key element in improving patient safety and care quality, optimizing organizational service systems, solving procedure problems, and promoting the sustainable high development of nursing disciplines (Ma et al., 2025; Wang et al., 2024; Abdelwahab Ibrahim El-Sayed et al., 2024). Nursing innovation refers to the process in which nurses create, develop, and implement new ideas, methods, and technologies within clinical practice, thereby introducing novel approaches to improve care delivery (Hashemian Moghadam et al., 2024). Such innovation helps to reduce potential safety risks, improve patient experience, and enhance the professional value of nurses. However, nurses’ innovative behavior is often restricted by heavy workloads, insufficient supportive resources, lack of professional motivation, and limited innovative capacity in clinical settings (Shi et al., 2026; Wang et al., 2026; Zhou et al., 2026). Therefore, exploring the influencing factors and internal mechanisms of nurses’ innovative behavior has become an important issue for nursing management.

As a vital contextual factor, perceived organizational support reflects the extent to which organizations value employees’ contributions and care about their well-being (Wang et al., 2026; Ni et al., 2026). Based on organizational support theory and social cognitive theory (Bandura, 1986; Rhoades and Eisenberger, 2002; Caesens and Stinglhamber, 2020), a supportive organizational environment can provide nurses with emotional encouragement, resource provision, policy support, and career development opportunities. When nurses perceive high levels organizational support, they tend to exhibit more positive work attitudes, stronger work engagement, and greater willingness to conduct exploratory and innovative behaviors (Li H. et al., 2026; Larsman et al., 2024). Although previous studies have confirmed the positive effect of perceived organizational support on nurses’ innovative behavior, most studies have focused on direct effect analysis, and the multiple mediating pathways through which perceived organizational support promotes nurse innovation remain unexplored (Wang et al., 2026; Zhou et al., 2023). Existing research regarding mediating mechanisms has preliminarily been derived from corporate employee samples, with limited empirical validation among clinical nurses (Wang, 2025). Therefore, clarifying the underlying mechanism by which perceived organizational support influences nurses’ innovative behavior is essential to provide a theoretical basis for future intervention research.

In the context of digital health transformation, digital competence has become an indispensable skill for nurses (Jimenez et al., 2020; Nazeha et al., 2020). It includes the ability to acquire professional nursing information, operate intelligent medical devices, analyze clinical data, and apply digital technologies to solve practical problems (Ochoa Pacheco and Coello-Montecel, 2023; Hammarén et al., 2025). Nurses with higher digital competence are more inclined to break through traditional working patterns, integrate emerging technologies into clinical practice, and generate innovative ideas (Wei et al., 2025). Previous studies have consistently shown that perceived organizational support can effectively promote nurses’ digital competence (Jameela et al., 2026; Alhaiti, 2025; Laakkonen et al., 2024). However, the association between digital competence and innovative behavior remains poorly understood in clinical nursing samples. Relevant evidence is restricted to nursing education, where digital competence has been found to significantly predict pedagogical innovation among nursing educators (Asal et al., 2025). Therefore, further investigation is warranted to examine whether digital competence serves as a mediating variable linking perceived organizational support to innovative behavior among clinical nurses.

As a positive psychological state characterized by heightened vitality and continuous learning, work thriving reflects individuals’ intrinsic motivation and ongoing growth at work (Gretchen Spreitzer et al., 2005). From the perspective of social embeddedness theory (Gretchen Spreitzer et al., 2005), individual psychological states and behaviors are not isolated, but are deeply embedded within organizational and social environment (Gretchen Spreitzer et al., 2005). In this framework, perceived organizational support represents a critical social-contextual resource that shapes nurses’ level of work thriving. Specifically, emotional care, resource guarantee, and a supportive work climate embed nurses in a positive organizational network, thereby enhancing their sense of vitality at work and motivation for continuous learning, which are the core connotations of work thriving (Li Y. et al., 2026). Existing research indicates that supportive organizational environments can effectively enhance employees’ work thriving (Terry et al., 2025; Li Y. et al., 2026). Nurses with a higher level of work thriving tend to exhibit greater proactive work engagement, greater creative thinking, and more proactive and constructive behaviors in clinical practice (Ge et al., 2025; Slåtten et al., 2025). Although prior studies have confirmed that perceived organizational support promotes employees’ work thriving (Wang et al., 2026; Gabay et al., 2025), and that work thriving is also closely associated with positive work behaviors (Slåtten et al., 2025), the potential mediating role of work thriving in the relationship between perceived organizational support and nurses’ innovative behavior has not been empirically examined in the context of clinical nursing. Given the important guiding significance of social embeddedness theory for understanding how contextual factors shape psychological states and subsequent behaviors, exploring work thriving as a mediating bridge will deepen understanding of nurses’ innovative behavior and provide targeted nursing management strategies.

Thus, this study aimed to (1) verify the relationship among perceived organizational support, digital competence, work thriving, and innovative behavior among nurses, (2) explore whether digital competence and work thriving play parallel mediating roles between perceived organizational support and innovative behavior among nurses, and (3) provide empirical evidence and practical implications for designing competency-based and psychological interventions to enhance nurses’ innovative behavior. Therefore, we propose the following hypothesis:

*Hypothesis 1*: Perceived organizational support is positively associated with innovative behavior among nurses.

*Hypothesis 2*: Digital competence mediates the relationship between perceived organizational support and innovative behavior among nurses.

*Hypothesis 3*: Work thriving mediates the relationship between perceived organizational support and innovative behavior among nurses.

**Figure 1:**
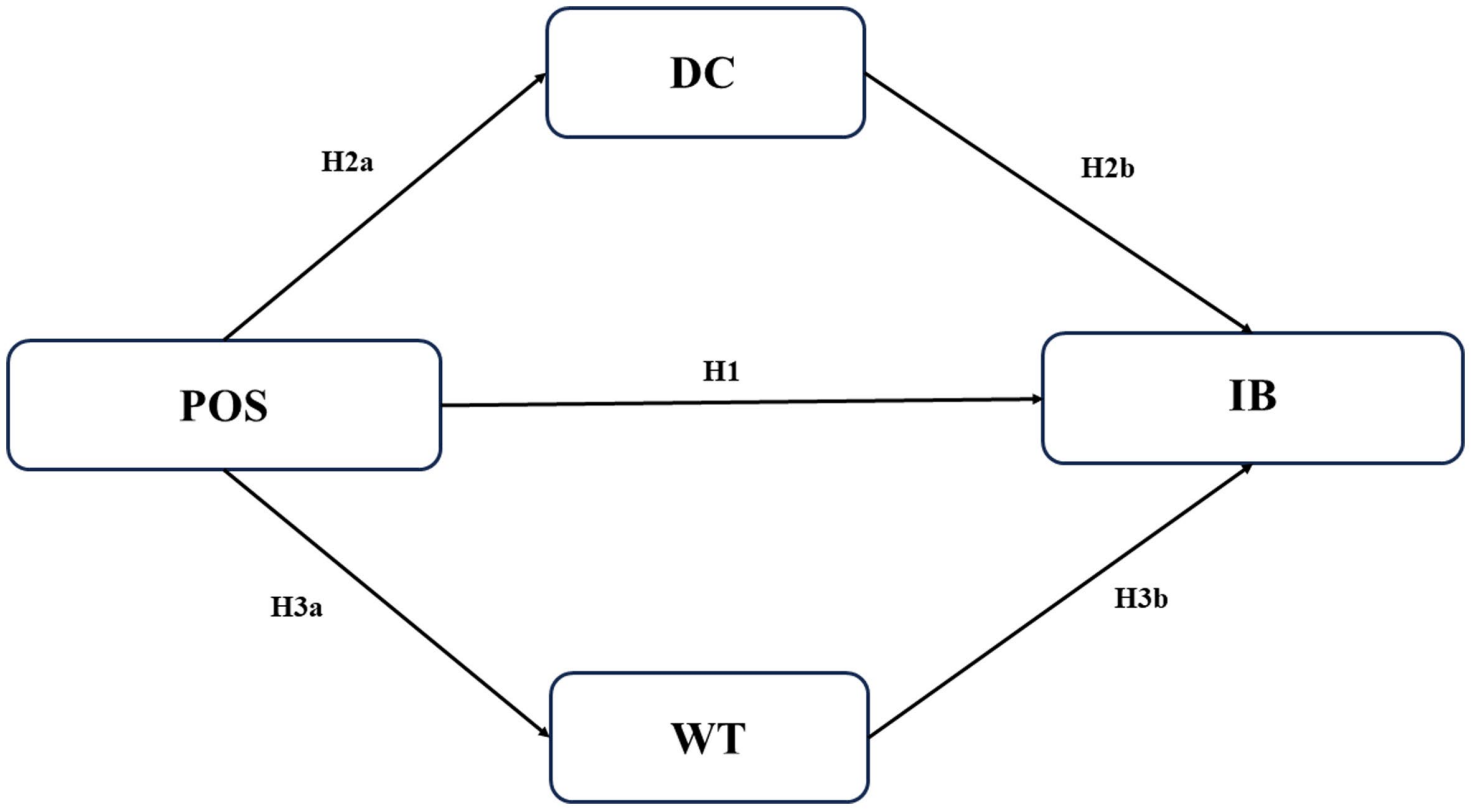
The theoretical model of this study. POS, perceived organizational support; DT, digital competence; WT, work thriving; IB, innovative behavior.

The theoretical model was shown in Figure 1.

## 2 Methods

### 2.1 Participants

A cross-sectional study was conducted between July and August 2025 from five hospitals in Chongqing. We utilized ANOVA with *α* = 0.05, *β* = 0.2 (80% power), an effect size (*f* = 0.25). Considering a 20% invalid rate, the minimum sample size was determined to be 227 participants (Faul et al., 2009). The sample size of this study satisfies the minimum required sample size for the statistical analyses. The inclusion criteria were as follows: (1) registered nurses; (2) nurses who were working in front-line; had at least 1 year of working experience; (4) Nurses who agreed to participate in the anonymous survey. The exclusive criteria were as follows: nurses who went out for study or vacation; (2) nurses who were pregnant.

### 2.2 Measures

All instruments were administered in their validated Chinese’s forms. Unless otherwise specified, psychometric information refers to the published Chinese validation studies for each instrument. After introducing each measurement scale, all core variables of this study were systematically sorted out. Table 1 presents the main variables, operational definitions, coding rules, corresponding scale items and supporting references, so as to clarify the connotation and measurement basis of each variable.

**Table 1:** Details of variables.

Variables	Operational definition	Coding	Items	References
Perceived organizational support	Nurses’ overall perception that the organization attaches importance to their work contributions, cares about their well-being, and provides emotional and working support in clinical practice	5-point Likert scale, ranging from 1 (completely disagree) to 5 (completely agree)	13	Hongmei and Hui (2009)
Digital competence	Nurses’ digital competence refers to the comprehensive ability to proficiently apply digital technologies in nursing practice, It reflects nurses’ capacity to skillfully use hospital digital systems, intelligent nursing equipment, and online platforms to complete daily nursing work efficiently and professionally	5-point Likert scale, ranging from 1 (completely disagree) to 5 (completely agree)	15	Zeyu et al. (2024)
Work thriving	A positive psychological experience of nurses at work, including two core components: learning vitality and emotional vitality, reflecting positive energy and active working status during nursing service	7-point Likert scale, ranging from 1(completely inconsistent) to 7 (completely consistent)	10	Lianping et al. (2020)
Innovative	Nurses’ innovative behavior refers to the behavioral tendency of nurses to actively put forward new thoughts, optimize nursing procedures, and adopt innovative strategies to solve practical clinical nursing problems	5-point Likert scale, ranging from 1 (strongly disagree) to 5 (strongly agree)	20	Liming et al. (2021)

Prior to testing the parallel mediation model, a confirmatory factor analysis (CFA) was conducted to validate the four-scale measurement model used by AMOS 24.0. The model fit indices including *χ*^2^/df, RMSEA, CFI, TLI, and NFI were calculated. The acceptable criteria were set as *χ*^2^/df < 3.0, RMSEA < 0.08, and CFI, TLI, NFI > 0.90.

#### 2.2.1 Demographic information

A self-administered and structured questionnaire was developed to collect participants’ demographic characteristics, including gender, age, marital status, children number, educational level, hospital grade, employment type, department, professional title, work experience, and monthly income.

#### 2.2.2 Perceived organizational support scale

Perceived organizational support was assessed using the scale revised by Hongmei and Hui (2009). This scale consists two dimensions and 13 items, including emotional support (10 items) and instrumental support (3 items). It was rated on a 5-point Likert scale ranging from 1 (completely disagree) to 5 (completely agree), with total scores ranging from 13 to 65. Higher total scores represent higher levels of perceived organizational support. In this study, the Cronbach’s *α* coefficient of this scale was 0.987. Convergent validity was assessed using average variance extracted (AVE) and composite reliability (CR). The AVE values were 0.945, and the corresponding CR values was 0.972.

#### 2.2.3 Digital competence scale

Digital competence was evaluated using the scale developed by Zeyu et al. (2024). This scale contains 15 items across five dimensions, including digital technology competence (2 items), information and data literacy (3 items), digital communication and innovation (4 items), digital value and pursuit (2 items), and basic personality traits for digital usage (4 items). Responses were scored on a 5-point Likert scale from 1 (completely disagree) to 5 (completely agree), yielding a total score range of 5 to 75. Higher scores indicate better digital competence among nurses. In the present study, the Cronbach’s *α* coefficient for this scale was 0.978, AVE was 0.794, and CR was 0.951.

#### 2.2.4 Thriving at work scale

The Thriving at Work Scale was originally developed by Porath et al. (2011) and translated into Chinese by Lianping et al. (2020). This Scale comprises 10 items covering two dimensions: learning and vitality, with five items in each subscale. Responses were rated on a 7-point Likert scale ranging from 1 (completely inconsistent) to 7 (completely consistent), with total scores ranging from 7 to 70. Higher scores indicate a higher level of work thriving. In the current study, the Cronbach’s *α* coefficient of this scale was 0.774, AVE was 0.757, and CR was 0.862.

#### 2.2.5 Innovative behavior scale

The Innovative Behavior Scale was originally developed by Lukes and Stephan (2017) and adapted into Chinese by Liming et al. (2021). This scale includes 20 items across five dimensions: idea generation and search (six items), plan communication and implementation (five items), acquisition of human resources (three items), overcoming obstacles (three items), clinical application (three items). Participants responded on a 5-point Likert scale ranging from 1 (strongly disagree) to 5 (strongly agree), with total scores ranging from 20 to 100. Higher scores suggest more frequent and positive innovative behavior. The Chinese version has been validated among nurses and demonstrated good reliability and validity. In the present study, the Cronbach’s α coefficient of this scale was 0.982, AVE was 0.838, and CR was 0.963.

### 2.3 Procedure

Data were collected by an online tool called wenjuanxing, which is a widely-used and reliable platform in China. The electronic questionnaire link was distributed to directors of nursing departments and head nurses across the five hospitals to facilitate the survey process. Trained investigators were responsible for distributing questionnaires and providing necessary guidance to respondents. Prior to participation, a detailed introduction of the study purpose and procedures was provided to the participants. They were clearly informed of their right to voluntary participation and could withdraw or refuse to answer any questions at any time. All respondents were assured strict confidentiality, and the collected data would be used exclusively for scientific research purposes. Finally, a total of 450 questionnaires were distributed, and 414 valid responses were retrieved, representing a valid response rate 92%. Invalid questionnaires were excluded if they contained incomplete responses, showed regular response patterns, or were completed in less than 300 s.

### 2.4 Ethical considerations

The study was approved by the Ethics Committee of The First Affiliated of Chongqing Medical University (2024-035-01). Informed consent was obtained from all participants, who were informed of their right to withdraw from the study at any time without penalty. Participation in this research was entirely voluntary. The survey was administered anonymously, and all participants were assured that their responses would be kept strictly confidential.

### 2.5 Data analysis

SPSS 23.0 software was used for data analysis. Continuous variables were summarized as mean ± standard deviation (M ± SD), and categorical variables were reporting using frequency, percentage, and proportion. For normally distributed data, independent samples t-test and one-way analysis of variance (ANOVA) were performed to compare differences across groups accordingly. Additionally, confirmatory factor analysis (CFA) was conducted using AMOS 24.0 to verify the structural validity, convergent validity, and discriminant validity of the four-factor measurement model. Hayes’ PROCESS macro (Version 4.1) was applied to conduct parallel multiple mediation analysis, aiming to investigate the indirect effects of the independent variable on the dependent variable via the hypothesized mediators. The detailed results of the mediation analysis are presented in Table 2.

**Table 2:** Parallel mediation effects of digital competence and work thriving on the relationship between perceived organizational support and innovative behavior (*n* = 414).

Effect	*B*	*SE*	95%CI	*p*
Total effect (X → Y)	0.754	0.045	(0.666, 0.843)	<0.001
Direct effect (X → Y)	0.093	0.038	(0.018, 0.168)	<0.05
Indirect effect (X → M1 → Y)	0.549	0.056	(0.446, 0.665)	–
Indirect effect (X → M2 → Y)	0.113	0.029	(0.062, 0.173)	–
Total indirect effect	0.661	0.059	(0.555, 0.780)	–

X, perceived organizational support. M1, digital competence. Y, innovative behavior.

## 3 Results

### 3.1 Demographic characteristics and one-way multivariate analysis of covariance (innovative behavior)

A total of 414 nurses participated in this survey with a valid response rate of 99.5%. A majority of nurses were female, married, had a bachelor’s degree, contracted staff, working in general wards, and monthly income less than 10,000 (¥). About half of them were in 30–40 years old, working in tertiary A hospitals, had junior or intermediate professional title. Almost one third of them had 1 child and 11–15 years work experience (Table 3).

**Table 3:** The demographic information and univariate analysis of innovative behavior among nurses (*n* = 414).

Variables	Category	*N* (%)	M ± S	*t/F*	*p*	*Post hoc* comparison
Gender				−0.738	0.461	
	Male	9 (2.17)	80.44 ± 11.01			
	Female	405 (97.83)	83.54 ± 12.48			
Age				1.629	0.182	
	≤30	145 (35.02)	82.55 ± 12.68			
	30 ~ 40	217 (52.42)	83.26 ± 12.44			
	41 ~ 50	42 (10.14)	87.00 ± 11.05			
	>50	10 (2.42)	86.60 ± 13.33			
Marital status				1.315	0.270	
	Unmarried	81 (19.57)	81.47 ± 11.69			
	Married	314 (75.85)	83.94 ± 12.62			
	Divorced and widowed	19 (4.59)	84.26 ± 12.36			
Children number				2.366	0.095	
	0	113 (27.29)	81.31 ± 11.96			
	1	152 (36.71)	84.29 ± 13.14			
	≥2	149 (35.99)	84.28 ± 11.95			
Educational level				1.923	0.147	
	Junior college’s	48 (11.59)	81.19 ± 10.96			
	Bachelor’s	358 (86.47)	83.91 ± 12.61			
	Master’s and above	8 (1.93)	77.63 ± 11.76			
Hospital grade						
	Tertiary A^a^	201 (48.55)	84.71 ± 11.69	4.157	**0.016**	**a > b > c**
	Tertiary B^b^	148 (35.75)	83.48 ± 13.52			
	Secondary^c^	65 (15.70)	79.63 ± 11.52			
Employment type				1.242	0.215	
	Permanent	81 (19.57)	85.01 ± 14.20			
	Contracted	333 (80.43)	83.10 ± 11.97			
Department				5.798	**0.001**	**d > b > a > c**
	General wards^a^	331 (79.95)	83.19 ± 12.56			
	Critical care departments^b^	47 (11.35)	85.81 ± 10.13			
	Anesthesiology and operating room^c^	24 (5.80)	77.33 ± 12.62			
	Auxiliary departments^d^	12 (2.90)	94.33 ± 8.94			
Professional title				1.614	0.200	
	Junior	202 (48.79)	82.64 ± 12.68			
	Intermediate	192 (46.38)	83.94 ± 12.16			
	Senior and above	20 (4.83)	87.45 ± 12.33			
Work experience (year)				3.298	**0.011**	**e > d > b > c > a**
	1 ~ 5^a^	67 (16.18)	81.52 ± 12.19			
	5 ~ 10^b^	107 (25.85)	83.03 ± 12.30			
	11 ~ 15^c^	164 (39.61)	82.48 ± 12.83			
	16 ~ 20^d^	52 (12.56)	87.63 ± 10.90			
	>20^e^	24 (5.80)	88.71 ± 11.80			
Monthly income (¥)				0.275	0.760	
	≤10,000	331 (79.95)	83.30 ± 12.14			
	10,001 ~ 20,000	67 (16.18)	83.85 ± 13.62			
	>20,000	16 (3.86)	85.50 ± 12.44			

Bold values denote *p*-values.

Significant differences were observed between nurses across multiple variables, including hospital grade, department, and work experience (Table 3).

### 3.2 Scores of perceived organizational support, digital competence, work thriving and innovative behavior among nurses

Table 4 showed that mean scores of perceived organizational support, digital competence, work thriving and innovative behavior among nurses were 4.23 ± 0.81, 4.15 ± 0.66, 5.39 ± 0.85, and 4.17 ± 0.62, respectively.

**Table 4:** Scores of perceived organizational support, digital competence, work thriving and innovative behavior among nurses (*n* = 414).

Variables	Dimensions	Item	M ± SD (dimensions)	M ± SD (items)
Perceived organizational support		13	55.01 ± 10.47	4.23 ± 0.81
	Emotional support	10	42.15 ± 8.21	4.22 ± 0.82
	Instrumental support	3	12.92 ± 2.37	4.31 ± 0.79
Digital competence		15	62.30 ± 9.92	4.15 ± 0.66
	Digital technology competence	2	8.14 ± 1.50	4.07 ± 0.75
	Information and data literacy	3	12.27 ± 2.27	4.09 ± 0.76
	Digital communication and innovation	4	16.54 ± 2.83	4.14 ± 0.71
	Digital value and pursuit	2	8.53 ± 1.35	4.27 ± 0.68
	Basic personality traits for digital usage	4	16.83 ± 2.73	4.21 ± 0.68
Work thriving		10	53.94 ± 8.51	5.39 ± 0.85
	Learning	5	27.20 ± 4.42	5.44 ± 0.88
	Vitality	5	26.74 ± 4.67	5.35 ± 0.93
Innovative behavior		20	83.47 ± 12.44	4.17 ± 0.62
	Idea generation and search	6	25.41 ± 3.73	4.24 ± 0.62
	Plan communication and implementation	5	20.83 ± 3.31	4.17 ± 0.66
	Acquisition of human resources	3	12.20 ± 2.19	4.07 ± 0.73
	Overcoming obstacles	3	12.64 ± 1.99	4.21 ± 0.66
	Clinical application	3	12.40 ± 2.05	4.13 ± 0.68

M, Mean; SD, Standard deviation.

### 3.3 Correlations between perceived organizational support, digital competence, work thriving and innovative behavior among nurses

Table 5 indicated that all variables were moderately to strongly and significantly positively correlated with each other. The strongest association was observed between digital competence and innovative behavior (*r* = 0.871, *p* < 0.01).

**Table 5:** Correlation coefficients between perceived organizational support, digital competence, work thriving and innovative behavior among nurses (*n* = 414).

Variables	1	2	3	4	5	6	7	8	9	10	11	12	13	14	15	16	17	18
1. Perceived organizational support	1																	
2. Emotional support	0.997**	1																
3. Instrumental support	0.966**	0.945**	1															
4. Digital competence	0.640**	0.636**	0.624**	1														
5. Digital technology competence	0.532**	0.531**	0.513**	0.871**	1													
6. Information and data literacy	0.587**	0.587**	0.563**	0.945**	0.838**	1												
7. Digital communication and innovation	0.603**	0.598**	0.595**	0.966**	0.830**	0.937**	1											
8. Digital value and pursuit	0.628**	0.621**	0.627**	0.891**	0.680**	0.759**	0.811**	1										
9. Basic personality traits for digital usage	0.608**	0.606**	0.590**	0.927**	0.721**	0.794**	0.838**	0.899**	1									
10. Work thriving	0.615**	0.612**	0.599**	0.640**	0.560**	0.590**	0.621**	0.573**	0.600**	1								
11. Learning	0.563**	0.562**	0.539**	0.586**	0.518**	0.543**	0.571**	0.529**	0.541**	0.934**	1							
12. Vitality	0.589**	0.584**	0.583**	0.613**	0.530**	0.562**	0.593**	0.544**	0.583**	0.941**	0.756**	1						
13. Innovative behavior	0.635**	0.629**	0.630**	0.871**	0.715**	0.780**	0.839**	0.821**	0.847**	0.665**	0.612**	0.633**	1					
14. Idea generation and search	0.586**	0.579**	0.585**	0.847**	0.672**	0.742**	0.814**	0.816**	0.843**	0.634**	0.584**	0.604**	0.949**	1				
15. Plan communication and implementation	0.639**	0.632**	0.635**	0.833**	0.686**	0.744**	0.798**	0.800**	0.810**	0.622**	0.579**	0.587**	0.958**	0.887**	1			
16. Acquisition of human resources	0.579**	0.578**	0.561**	0.797**	0.690**	0.744**	0.774**	0.697**	0.751**	0.643**	0.585**	0.620**	0.909**	0.809**	0.827**	1		
17. Overcoming obstacles	0.598**	0.591**	0.597**	0.788**	0.642**	0.698**	0.756**	0.770**	0.766**	0.589**	0.543**	0.560**	0.929**	0.852**	0.876**	0.801**	1	
18. Clinical application	0.558**	0.553**	0.553**	0.785**	0.654**	0.711**	0.766**	0.719**	0.752**	0.618**	0.567**	0.592**	0.926**	0.822**	0.854**	0.866**	0.849**	1

**p < 0.01.

### 3.4 Independent factors influencing factors of innovative behavior

Stepwise multiple regression analysis revealed that perceived organizational support, work thriving, and digital competence were significant independent predictors of innovative behavior (Table 6).

**Table 6:** Multiple linear regression analysis of innovative behavior among nurses (*n* = 414).

Variables	*B*	*β*	*t*	*p*
Constant	7.435	-	3.083	0.002
Hospital grades	0.573	0.034	1.423	0.155
Department	0.179	0.010	0.438	0.661
Work experience	0.250	0.022	0.919	0.359
Perceived organizational support	0.093	0.078	2.409	**0.016**
Work thrives	0.225	0.154	4.785	**<0.001**
Digital competence	0.910	0.725	21.912	**<0.001**

*R*^2^ = 0.783, adjust *R*^2^ = 0.779, *F* = 244.086, VIF<5, no significant covariance in the independent variables and normally distributed residuals. Bold values denote *p*-values.

### 3.5 Confirmatory factor analysis (CFA)

**Figure 2:**
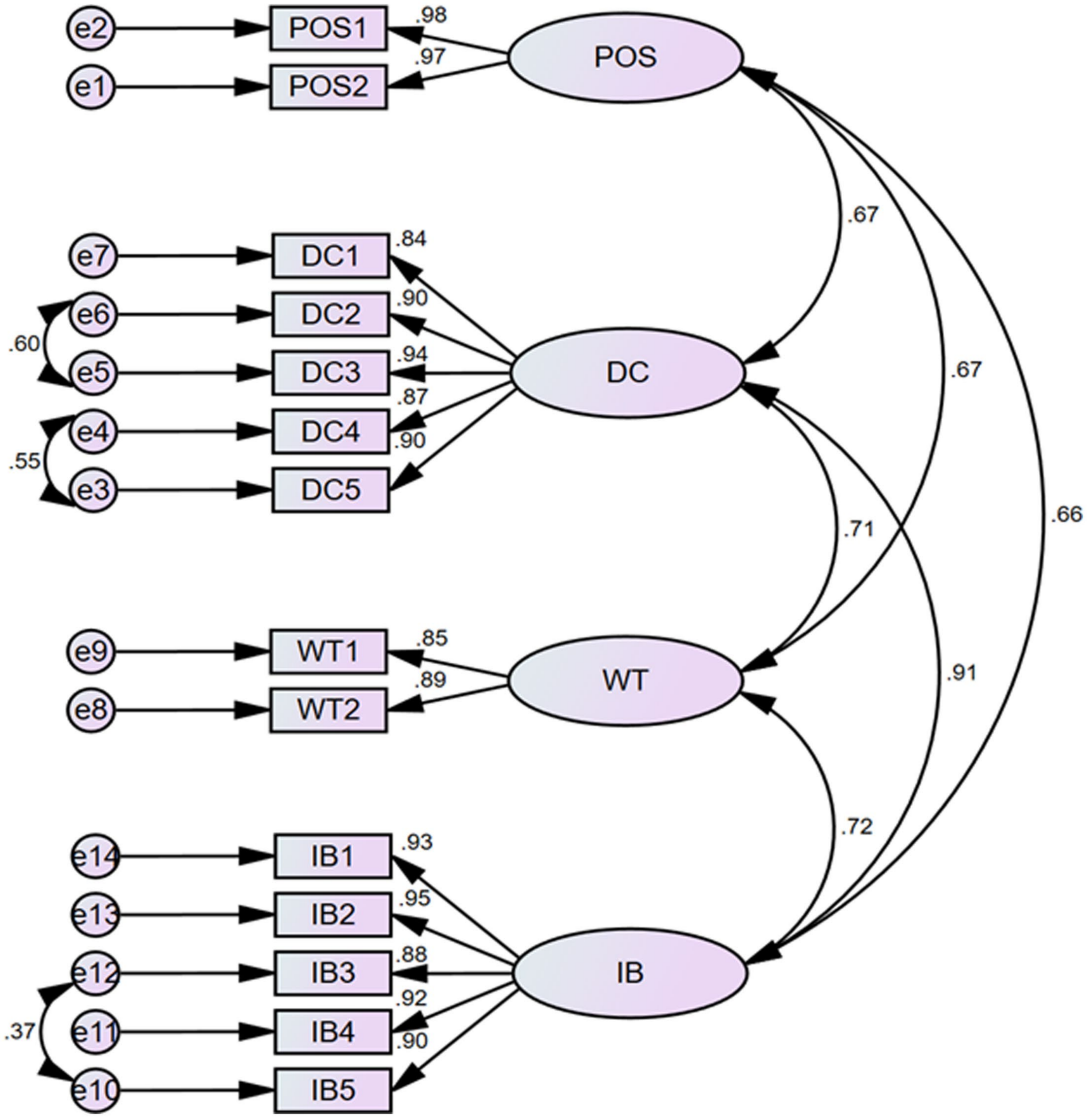
Confirmatory factor analysis model of the four-factor measurement model. POS, perceived organizational support; DT, digital competence; WT, work thriving; IB, innovative behavior.

The fit results of the four-factor measurement model are shown in Table 7, and the standardized path coefficients are presented in Figure 2. All indices indicating that the four latent variables had good validity. Specifically, the model demonstrated acceptable fit: *χ*^2^/df = 4.273, RMSEA = 0.089, NFI = 0.962, CFI = 0.971, TLI = 0.961. Convergent and discriminant validity were further assessed. Convergent validity was supported as all AVE values exceeded 0.50 (range: 0.757–0.945) and all CR values exceeded 0.70 (range: 0.862–0.972). Discriminant validity was examined using the Fornell-Larcker criterion. The square root of AVE for each construct exceeded its correlations with other constructs, confirming that the four factors are empirically distinct.

**Table 7:** Model fit indices of the confirmatory factor analysis.

Items	χ^2^/df	RMSEA	NFI	TLI	CFI
Reference value	<5.0	<0.08	>0.9	>0.9	>0.9
Including e1–e16 covariance	4.273	0.089	0.962	0.961	0.971

To test potential common method bias, we compared the fit of the four-factor baseline model and the single-factor alternative model. The four-factor model exhibited acceptable model fit: *χ*^2^/df = 4.273, RMSEA = 0.089, NFI = 0.962, CFI = 0.971, TLI = 0.961. In contrast, the single-factor model showed poor fit: *χ*^2^/df = 24.693, RMSEA = 0.240, NFI = 0.755, CFI = 0.762, TLI = 0.710. The substantial difference in model fit indicated that the four-factor structure was empirically distinct, and common method bias was not a serious concern in this study.

### 3.6 Mediating analysis

**Figure 3:**
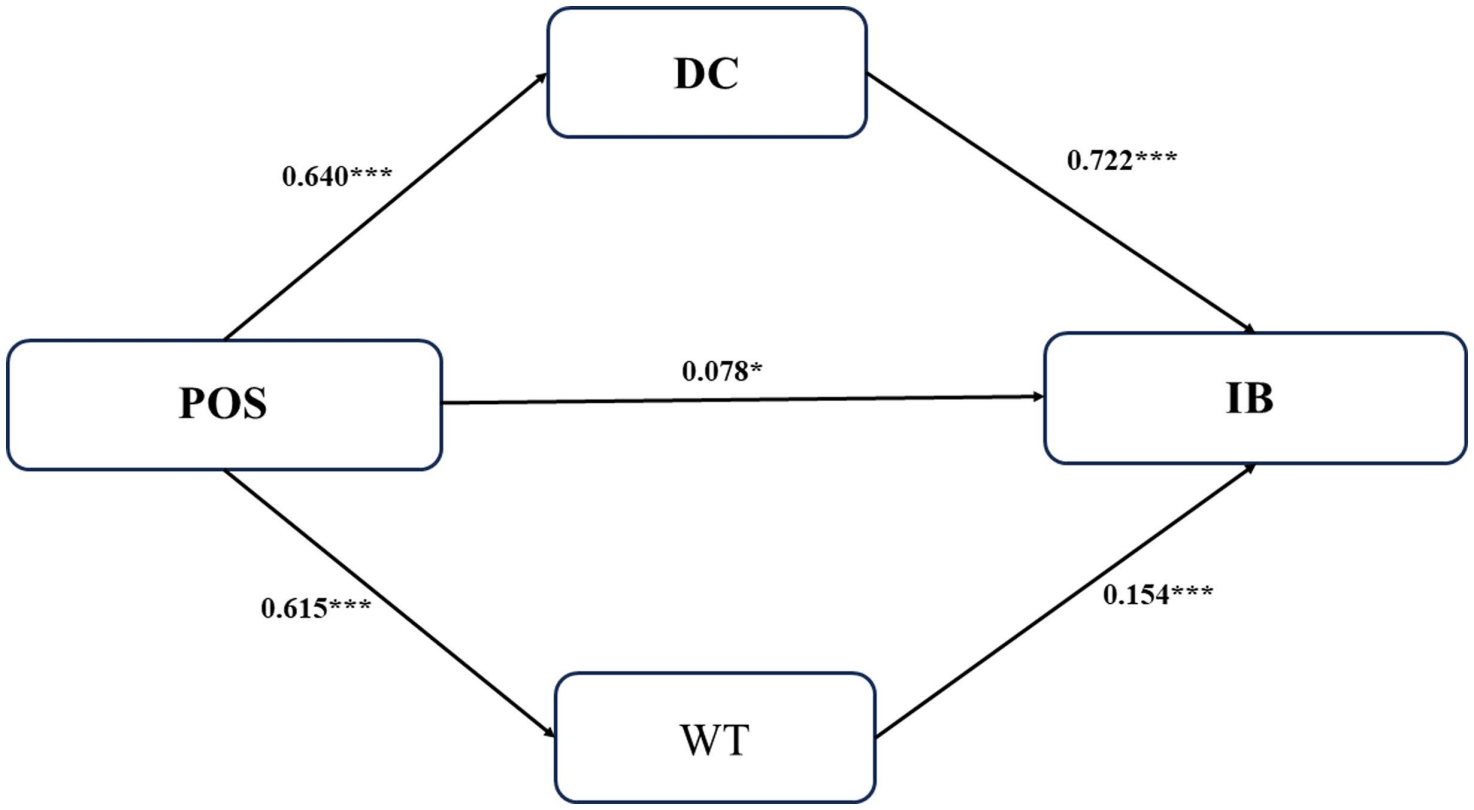
The mediated model. ***p < 0.001, *p < 0.05. POS, perceived organizational support; DT, digital competence; WT, work thriving; IB, innovative behavior.

We employed Model 4 of Hayes’ PROCESS macro to examine the parallel mediating roles of digital competence and work thriving in the relationship between perceived organizational support and innovative behavior. Figure 3 presents the standardized path coefficients of the model, and Table 2 summarizes the total, direct, and indirect effects.

#### 3.6.1 Path 1

The total effect of perceived organizational support on innovative behavior was significant and positive (*β* = 0.635, *t* = 16.686, *p* < 0.001). Perceived organizational support significantly and positively predicted digital competence (*β* = 0.640, *t* = 16.896, *p* < 0.001), and digital competence significantly and positively predicted innovative behavior (*β* = 0.722, *t* = 21.918, *p* < 0.001). The direct effect of perceived organizational support on innovative behavior remained significant after simultaneously controlling for both digital competence and work thriving (*β* = 0.078, *t* = 2.439, *p* = 0.015), confirming a partial mediating role of digital competence.

#### 3.6.2 Path 2

Perceived organizational support significantly and positively predicted work thriving (*β* = 0.615, *t* = 15.834, *p* < 0.001), and work thriving significantly and positively predicted innovative behavior (*β* = 0.154, *t* = 4.809, *p* < 0.001). The direct effect of perceived organizational support on innovative behavior remained significant after simultaneously controlling for both digital competence and work thriving (*β* = 0.078, *t* = 2.439, *p* = 0.015), confirming a partial mediating role of work thriving.

## 4 Discussion

The present study demonstrated that perceived organizational support not only exerted a significant direct effect on innovative behavior among clinical nurses, but also exerted an indirect influence through digital competence and work thriving. Specifically, digital competence and work thriving played parallel mediating roles in the relationship between perceived organizational support and innovative behavior.

### 4.1 Relationship between perceived organizational support and innovative behavior

The results indicated that perceived organizational support (X) was moderately and positively correlated with innovative behavior (Y) among nurses, supporting Hypothesis 1. This finding aligns with previous studies (Wang et al., 2026; Zhou et al., 2023), and is consistent with recent evidence reported by Bindel Sibassaha et al. (2025), who demonstrated that higher perceived organizational support was associated with more frequent innovative behavior. Conversely, nurses who engage more actively in innovative practices are also more likely to receive increased recognition and resource support from organizations. Further path analyses demonstrated that perceived organizational support exerted a significant direct predictive effect on innovative behavior in both mediation models. Specifically, in the parallel mediation model incorporating both digital competence and work thriving simultaneously, the direct effect of perceived organizational support on innovative behavior was *β* = 0.078. This finding indicated a statistically significant but relatively weak direct influence (Bandura, 1986). Perceived organizational support in this study comprised emotional and instrumental support (Saleh et al., 2025; Wei et al., 2026). On the one hand, emotional support provides nurses with professional recognition (Wei et al., 2026) and well-being (Sheng et al., 2023), alleviates occupational burnout (Luo et al., 2022) and work-related stress (Ali et al., 2022), and thus fosters active engagement in innovative practices (Saleh et al., 2025). On the other hand, instrumental support provides nurses with essential resources, training, institutional support, and a supportive climate for innovation (Ma et al., 2025; Zhang et al., 2022; Ge et al., 2025), creating practical conditions for nurses to implement innovative behaviors in clinical placement. Collectively, these findings support that higher levels of perceived organizational support are associated with more frequent innovative behavior among nurses.

### 4.2 The mediation role of digital competence

The results of this study showed that digital competence mediated the relationship between perceived organizational support and innovative behavior among nurses, which verified Hypothesis 2. The results is in line with the previous findings of Yang and Zhou (2022), who confirmed the mediating role of digital thinking positively moderates the relationship between perceived organizational support and innovative self-efficacy. Path analysis revealed that perceived organizational support (X) positively and significantly predicted digital competence (M1) (*β* = 0.640, *p* < 0.001), representing a moderate-to-strong effect size. In turn, digital competence (M1) exerted a strong positive predictive effect on innovative behavior (Y) (*β* = 0.722, *p* < 0.001). The completely standardized indirect effect was 0.462, with a 95% bootstrap confidence interval of [0.389, 0.533], which excluded zero, confirming the significance of the mediating effect. In addition, the direct effect of perceived organizational support (X) on innovative behavior (Y) remained significant (*β* = 0.078, *p* = 0.015), indicating that digital competence (M1) played a partial mediating role in this relationship.

These findings indicate that when nurses perceive stronger organizational support, including emotional and instrumental support, such as targeted upskilling, peer mentoring, and leadership engagement (Mikkonen et al., 2026), they are prone to develop more positive evaluations of their digital skills, information processing ability, and online communication capacity. These results have been confirmed in previous studies (Xu et al., 2025). Enhanced digital competence further encourages nurses to generate new ideas, optimize clinical procedures, and implement innovative practices in daily work, that is, a higher level of innovative behavior. Similar results were found by Wu et al. (2025), who suggested digital literacy positively predicted innovative behavior in Chinese medical students. The reason for this is that a supportive organizational climate has a positive effect on an individual’s skill development and cognitive appraisal. Positive appraisal not only improves an individual’s ability to apply digital skills in clinical work (Högstedt et al., 2024), but also enhances their confidence in exploring new working models (Lu et al., 2023) and boosts their persistence in implementing innovative practices (Wang et al., 2026). This pathway supports the social cognitive theory and extends previous studies by demonstrating that perceived organizational support and digital competence are important factors influencing innovative behavior in the nurse population.

### 4.3 The mediation of work thriving

The results of this study showed that work thriving mediated the relationship between perceived organizational support and innovative behavior among nurses, which verified Hypothesis 3. This result is consistent with the previous results of Jiao et al. (2026), who also found work thriving played a mediation role between trait mindfulness and innovative behavior among employees based on social cognitive theory. Similar results were found by Zhang et al. (2026), which showed work thriving was a mediation between various organizational factors and innovative behavior among company employees from three provinces in China. Perceived organizational support positively and significantly predicted work thriving (*β* = 0.615, *p* < 0.001), and work thriving in turn positively predicted innovative behavior (*β* = 0.154, *p* < 0.001). The completely standardized indirect effect was 0.095, with a 95% bootstrap confidence interval of [0.052, 0.146], excluding zero and verifying the significance of the mediating effect. The direct effect of perceived organizational support on innovative behavior remained significant (*β* = 0.078, *p* = 0.015), indicating that work thriving also functioned as a partial mediator.

The results of this study indicate that, when nurses perceive stronger organizational support, they tend to become more confident of work engagement, mainly performed as taking more time and energy of their continuous learning ability and workplace vitality (Zhai et al., 2023). This positive work engagement will promote their creative and innovative behavior in clinical workplace. Nurses who thrive at work tend to deepen their comprehension of clinical challenges via ongoing learning, embrace novel ideas and viewpoints, expand their thinking boundaries, and improve their ability to identify problems and develop solutions, which in turn fosters greater innovative behavior (Ge et al., 2025). The findings of the present study enrich the relevant research on the socially embedded model of thriving at work by demonstrating that work thriving is an important factor influencing the relationship between perceived organizational support and innovative behavior in the nurse population (Gretchen Spreitzer et al., 2005). It is consistent with the socially embedded model of thriving at work, which states that when individuals can perceive the value of their work in a supportive organizational context, this value enhances their motivation to engage in proactive behaviors, that is, it enhances their work thriving (Gretchen Spreitzer et al., 2005). As work thriving increases, individuals are willing to put in more effort when faced with clinical challenges and explore new solutions (Slåtten et al., 2025). This suggests that enhancing the work thriving of nurses can be used as one of the effective ways to improve their innovative behavior.

### 4.4 Theoretical implications

The present study constructed and verified a parallel multiple mediation model, revealing that perceived organizational support (X) can promote nurses’ innovative behavior (Y) both directly and indirectly through two distinct pathways: digital competence (M1) and work thriving (M2). Collectively, these two mediators accounted for a substantial proportion of the total effect of perceived organizational support on innovation, highlighting the complexity and multiplicity of underlying mechanisms.

From a theoretical perspective, on the one hand, consistent with social cognitive theory, perceived organizational support functions as an important environmental resource that cultivates nurses’ digital competence (M1), a core capability that enables them to translate supportive conditions into concrete innovative actions. On the other hand, in line with the social embeddedness model of work thriving, perceived organizational support embeds nurses in a positive work context that enhances work thriving (M2), a positive psychological state that motivates proactive, creative, and persistent engagement in innovative practices. Together, the cognitive pathway (M1) and the psychological pathway (M2) jointly explain how perceived organizational support promotes innovation.

Notably, the two mediators exhibited distinct effect magnitudes, with digital competence showing a relatively stronger indirect effect than work thriving. This difference suggests that in the era of digital health transformation, cognitive and technical capacity may serve as a more direct and powerful factor of innovative behavior among clinical nurses, while positive psychological states provide important supplementary motivation. Such findings enrich the literature by clarifying the relative importance of cognitive and psychological mechanisms in nursing innovation and offer an empirical foundation for designing multi-dimensional interventions to promote innovation in nursing practice.

## 5 Conclusion

This study used mediation analysis to examine the mediating roles of digital competence and work thriving in the relationship between perceived organizational support and innovative behavior among nurses, and explored the influencing factors and correlations among these variables. The findings revealed that hospital grade, department, and work experience were significant positive predictors of nurses’ innovative behavior. Specifically, digital competence and work thriving played parallel partial mediating roles between perceived organizational support and innovative behavior.

The study highlights that improving perceived organizational support, enhancing nurses’ digital competence, and promoting their work thriving are effective strategies to stimulate clinical innovation, providing empirical evidence for nursing management practice.

## 6 Limitations

Several limitations should be acknowledged. First, this study adopted a cross-sectional design, which precludes causal inferences about the relationships among perceived organizational support, digital competence, work thriving, and innovative behavior. Second, all data were collected via self-reported questionnaires, which may be subject to recall bias. Third, the sample was recruited from only a few regions and hospitals, which may limit the generalizability of the findings to other nursing populations. Fourth, this study only examined the mediating roles of digital competence and work thriving; other potential psychological or contextual variables were not included, which may provide a more comprehensive understanding of nurses’ innovative behavior.

## Data Availability

The original contributions presented in the study are included in the article/supplementary material, further inquiries can be directed to the corresponding author.
